# Resistance to chemotherapy

**DOI:** 10.1038/sj.bjc.6604663

**Published:** 2008-09-09

**Authors:** J Hofmann

**Affiliations:** 1 Biocenter, Division of Medical Biochemistry, Innsbruck Medical University, Fritz-Pregl-Strasse 3, Innsbruck A 6020, Austria

**Sir,**

I want to comment on a review by Raguz and Yagüe (2008) in a recent issue of *Br J Cancer*. The authors state correctly ‘although traditionally associated with the last stages of disease, recent findings with minimally transformed pre-tumourigenic primary human cells indicate that the ability to generate drug resistance arises early during the tumourigenic process and before the full transformation’. Here I try to explain why tumour cells can be resistant at the pre-tumourigenic stage and why in later stages of the disease.

Cancer cells may be intrinsically resistant because (i) as mentioned by the authors, the cancer stem cell hypothesis states that many, if not all, cancers contain a minority of stem cells. Stem cells are resistant to apoptosis, exhibit enhanced DNA repair activities and express membrane-bound ATP-binding cassette transporter (ABC) transporters that make them resistant to exogenous poisons (and chemotherapeutic agents) (Sakariassen *et al*, 2007; Styczynski and Drewa, 2007; Wicha *et al*, 2006). (ii) one can assume that a tumour exhibits similar sensitivity (or resistance) as the tissue from which the tumour originates. If the normal tissue and the tumour exhibit similar sensitivity, there is no therapeutic window, and at drug concentrations at which the tumour responds, normal tissue is damaged. In this case, the tumour resistance also exists already in minimally transformed pre-tumourigenic cells (intrinsic or inherent resistance). Therefore, tumour cells that respond to treatment must be more sensitive than the untransformed tissue from which the tumour cells originate. Thus one important question arises: what makes a tumour more sensitive than the tissue from which it originates, or in other words, what enables successful treatment of a certain percentage of tumours? One reason is that the tumour cells exhibit a different trait. For example, chronic myelogenous leukemia (CML) cells exhibit higher responsiveness to Bcr/Abl inhibitors, because Bcr/Abl is only expressed in tumour cells and drives tumour proliferation. The classical explanation for the success of traditional chemotherapy is that tumour cells are more sensitive because of their higher proliferation rate compared with most normal cells. However, there are several arguments against it. The first one is that if faster proliferation is the reason for sensitivity of tumour cells, at least all rapidly proliferating tumours should be sensitive to all or many of the traditional chemotherapeutic drugs. As we know, most tumours are only sensitive to one or several of the many antitumour drugs available. A second argument is that not all tumour cells are proliferating faster than non-transformed cells. For example, in the gastrointestinal tract, there are zones of fast proliferation of normal cells, and tumours derived from these cells need years to develop.

Therefore, at the starting point of tumourigenesis are stem cells or normal non-transformed cells, which exhibit a certain sensitivity. Let us assume that from these cells, tumour cells of the same sensitivity are generated. These tumour cells are classified as resistant because in case of such a tumour no therapeutic window is to be found. Thus, most of the tumours are resistant against most of the antitumour drugs. However, in certain tumours a majority of cells can be unusually sensitive to one or several anticancer drugs and, therefore, respond to therapy. These cells may become more sensitive to targeted therapy (by a new target) or to one or several of the traditional anticancer drugs (in many cases by unknown mechanisms) during tumourigenesis or as tumour cells. Upon treatment (i) sensitive cells are eliminated and the intrinsic resistant tumour cells survive and proliferate. (ii) Tumours can acquire resistance to the one or several drugs, to which they were sensitive and respond, in later stages by increased expression of ABC transporters, cytochrome 450, reduction of apoptosis, etc. This model can explain why resistance is to be found, on one side, in minimally transformed pre-tumourigenic cells, but on the other, it also can arise at later stages of the disease.
